# Chemical Enhancer: A Simplistic Way to Modulate Barrier Function of the Stratum Corneum

**DOI:** 10.15171/apb.2018.021

**Published:** 2018-06-19

**Authors:** Tasnuva Haque, Md Mesbah Uddin Talukder

**Affiliations:** ^1^Department of Pharmacy, East West University, A/2, Jahurul Islam City Gate, Aftab Nagar Main Rd, Dhaka-1212, Bangladesh.; ^2^Department of Pharmacy, BRAC University, 66 Bir Uttam AK Khandakar Road, Dhaka 1212, Bangladesh.

**Keywords:** Barrier function, Chemical enhancer, Drug delivery, Modification of skin, Stratum corneum

## Abstract

Human skin could be a prime target to deliver drugs into the human body as it is the largest organ of human body. However, the main challenge of delivering drug into the skin is the stratum corneum (SC), the outer layer of epidermis, which performs the main barrier function of the skin. Scientists have developed several techniques to overcome the barrier properties of the skin, which include other physical and chemical techniques. The most common and convenient technique is to use special formulation additives (chemical enhancers, CEs) which either drags the drug molecule along with it or make changes in the SC structure, thereby allowing the drug molecule to penetrate in to the SC. The main focus is to deliver drugs in the certain layers of the skin (for topical delivery) or ensuring proper percutaneous absorption (for transdermal delivery). However, skin drug delivery is still very challenging as different CEs act in different ways on the skin and they have different types of interaction with different drugs. Therefore, proper understanding on the mechanism of action of CE is mandatory. In this article, the effect of several CEs on skin has been reviewed based on the published articles. The main aim is to compile the recent knowledge on skin-CE interaction in order to design a topical and transdermal formulation efficiently. A properly designed formulation would help the drug either to deposit into the target layer or to cross the barrier membrane to reach the systemic circulation.

## Introduction

Since skin is the largest organ of the body, it could be a potential route to deliver drugs into the body. However, barrier property of the outer layer of the skin (stratum corneum, SC) limits the delivery of all types of drug in skin. Topical and transdermal formulations are delivered through the skin, targeting different layers of the skin and systemic circulation, respectively. Topical formulation delivers therapeutically effective concentration of a compound in the specific layer of the skin, to impart a local effect. As for example, sunscreen targets the outer layer of the skin,^1^ topical analgesic aims dermal-epidermal layer to reach cutaneous nociceptors,^2^ topical antifungals to viable epidermis,^3^ etc. In order to reach the specific layer of the skin and systemic circulation, a drug molecule must cross the SC and this only possible if barrier property of the skin is overcome. Chemical enhancers (CEs) are chemical agents which modify the SC barrier function and thereby allow molecules to penetrate into the skin. However, the penetration abilities of CEs changes as different CE interact with drug or skin differently. This article aims to summarize the recent findings on some commonly used CEs so that their incorporation into the formulation can develop more effective topical or transdermal or cosmetic products.

### Anatomy of skin

**Figure 1:**
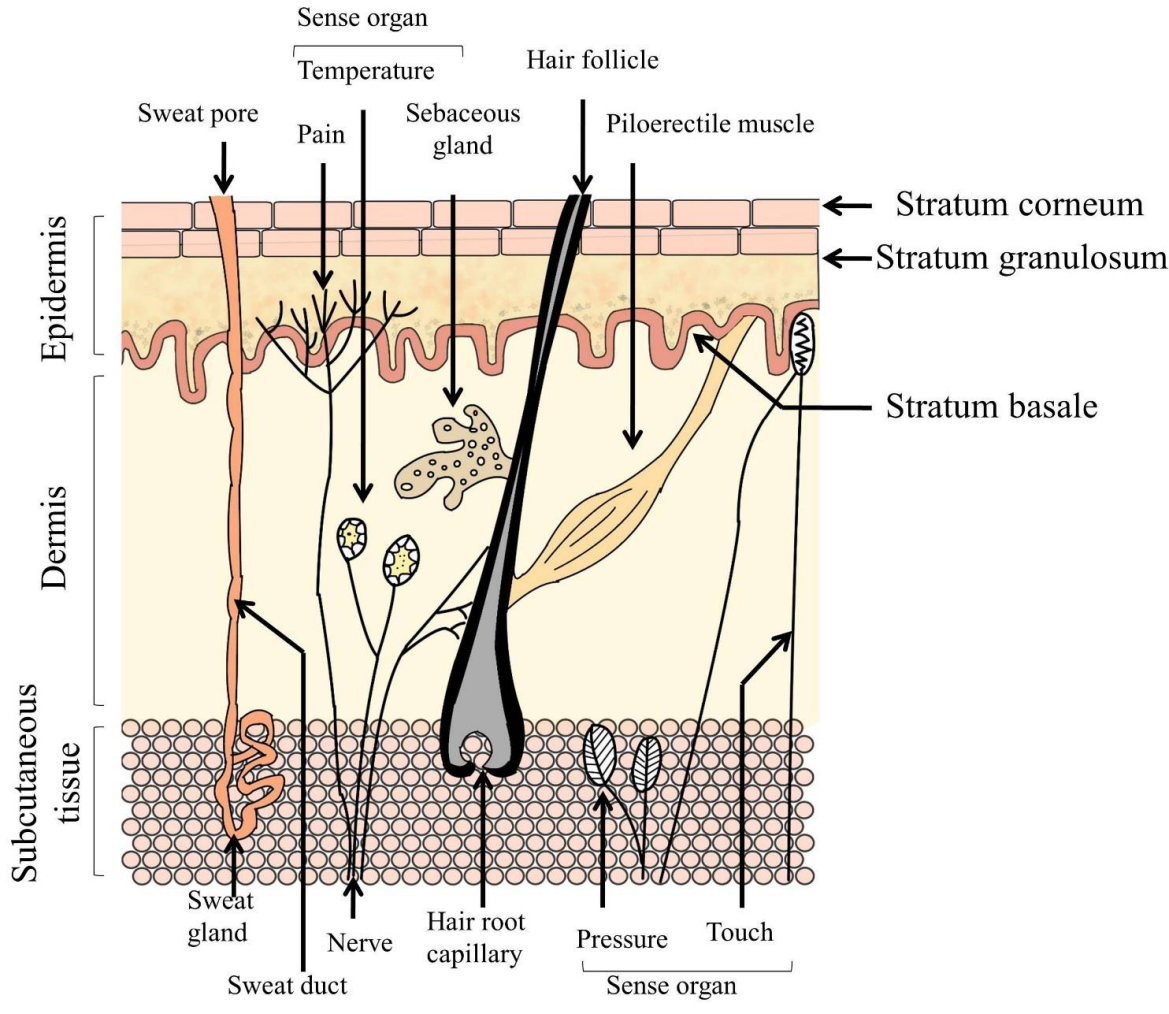
Structure of human skin

Skin has primarily three layers – epidermis (outer layer), dermis (middle layer) and subcutaneous tissue (bottom layer) (Figure 1).^4^ Epidermis contains five different cell strata. From outside to inside, these are stratum corneum (SC), stratum lucidum, stratum granulosum, stratum spinosum and stratum basale. The dermis consists of collagen fibrils and elastic connective tissues.^5^ This layer also contains mast cells, macrophages, lymphocytes and melanocytes.^6^ Immune and inflammatory responses are provided by the mast cells.^7^ Blood vessels, nerves and skin appendages (sweat and sebaceous glands) are also present in this layer. Because of the structural composition, this layer does not offer the same resistance to drugs as the SC. However, reduced permeation of lipophilic drugs may be observed in this layer.^7^ In the dermis, there are some sensory receptors such as thermoreceptors which sense temperature, nociceptors which sense pain and some mechanoreceptors which sense touch and pressure. The mechanoreceptors consist of Messiner’s corpuscles and Pacinian corpuscles which recognize light touch and pressure, respectively^6^ (Figure 1). The subcutaneous tissue is the inner layer containing fat cells interconnected by collagen and elastin fibres. This layer produces and stores large quantities of fat. It also protects the body from mechanical shock and stores large quantities of calories.^5,7^ There are several appendages present in the dermis and epidermis of human skin, such as eccrine and apocrine sweat glands, sebaceous glands, hair follicles and nails (Figure 1).

### Stratum corneum (SC), the main barrier of the skin

The SC is the outermost layer of epidermis having a heterogeneous structure and composed of 70 to 80% protein (keratin) and lipid.^7^ It provides the main barrier function of the skin. The SC is composed of 10 to 15 layers of compressed corneocytes present in the SC.^5,8^ Between the SC corneocytes different types of lipids are present.^9^ If the SC is picturized as a brick wall, the corneocytes are the ‘bricks’ present in a ‘mortar’ (or intercellular lipid matrix).^10^ Desmosomes are the connectors between the corneocytes. The corneocyte is surrounded by a protein-lipid polymeric envelope.^7^ The corneocytes are rigid because of the envelope.^11^ The intercellular space between corneocytes is filled with multiple lipid lamellae. The lamellae consist of ceramides, cholesterol, cholesterol esters, cholesterol sulphate and free fatty acids.^7,12^ The lipid lamellae are arranged horizontally to the surface of the corneocytes.^13,14^ Intercellular lipids act as shields to prevent water loss from the body.^15^ If the lipid from the SC is extracted, the lipid from the SC enhanced the water loss faster compared with non-extracted skin.^16^ Thus, intercellular lipid lamellae are very important for the barrier function of the SC and also help in cohesion between corneocytes.^17^ The SC also contains approximately 15 to 20% water mainly associated with keratin^7^ and a small amount in the polar head group of the intercellular space.^18^ The lower water loss and higher barrier function of the SC is because of the unique composition, especially due to the intercellular lipids and corneocyte envelope.^18,19^ The epidermis undergoes a differentiation process in which generation of the SC takes place. The process starts at the stratum basale and cells migrate upwards to the SC and it usually takes 2 to 3 weeks.^6^

### Routes of permeation in the SC

**Figure 2:**
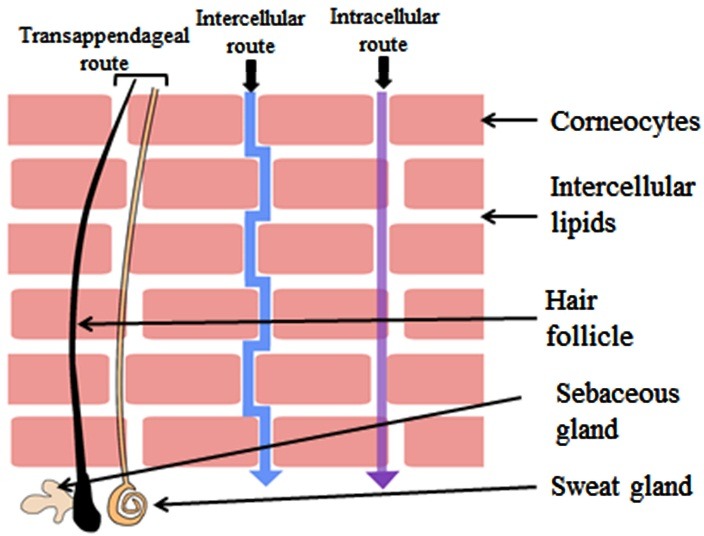
Routes of penetration of a molecule across the SC

Diffusion is the principle mechanism by which the permeation of a permeant across human skin takes place.^20^ A solute can diffuse through the skin by three main routes: the transappendageal route, the intracellular and intercellular route (Figure 2). Permeation through the transappendageal route is known as the permeation via the hair follicles, sebaceous and sweat glands. Appendageal transport provides an easier path of diffusion in parallel to the transepidermal route (intra- and intercellular routes). However, the skin appendages have very low surface area (only 0.1% of the total skin surface area).^21^ In addition, permeation of drugs is not direct in the sweat and sebaceous glands. Sweat moves in the reverse direction of the permeant in sweat gland. Moreover, permeation of only hydrophilic molecules is not possible in sebaceous glands as it has lipid-rich sebum.^22^ However, the transappendageal route can be vital for ions and large polar molecules which do not freely cross the SC.^23-25^

Transepidermal pathway is the route which includes intra- and intercellular permeation. If the SC structure is considered as ‘brick and mortar’, the intracellular is the shortest route through the layers of corneocytes and its surrounding intercellular lipid matrix. When such penetration takes place in a tortuous way via the intercellular lipid matrix, it is called the intercellular route. At first it was believed that hydrophilic drugs preferentially diffuse through the intracellular region and hydrophobic drugs through the intercellular matrix^21^. In both cases, the compound has to penetrate the intercellular lipid. However, later it was found that the intercellular route was the predominant pathway for permeation of most drugs through the human SC.^26-29^ Diffusion of a penetrant through the intracellular route requires undergoing via a series of partitioning and diffusion stages in and out of the relatively hydrophilic corneocytes, lipid envelope surrounding the corneocytes and the intercellular matrix. Whereas, the penetrant needs to take a tortuous route consisting of alternating structures of bilayers (containing both aqueous and lipid domains) in the intercellular route. In this route of permeation, a penetrant passes through a 50-times longer path length compared with the total thickness of the SC. A penetrant also has to undergo sequential partition and diffusion through the aqueous and lipid domains of the intercellular matrix.^22^

### Permeation of drug molecule across the skin

The percutaneous absorption of a solute involves a series of transport processes which is mainly determined by the solubility and diffusivity of the solute. The solubility of a solute in a solvent is determined by the solvent-solute interaction. Firstly, the solute requires to be solubilised into the outermost lipid layer of SC and then diffuses through it. These processes are affected by the CE-skin and solute-skin interactions. During this process the solute may also permeate into the corneocytes. In the next stage, again a series of partition followed by diffusion takes place in the viable epidermis and in the papillary dermis. Absorption of solute by the capillary plexus followed by distribution into the systemic circulation occurs in the papillary dermis. Being the prominent pathway, hydrophilic molecules permeate through the polar head groups and hydrophobic molecules permeate via the lipid chains of the bilayer regions of the intercellular route.^30^

### Drug-CE-skin interactions

**Figure 3:**
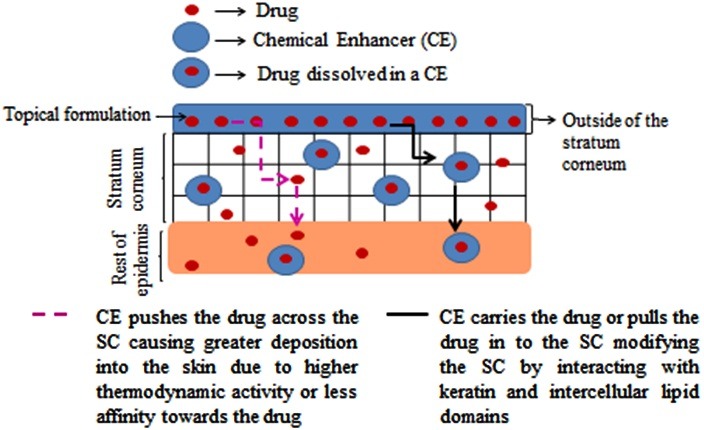
Interaction of CEs with the stratum corneum (‘Push-pull’-effect).

After applying a topical formulation on the surface of the skin, drug - CE, CE -skin and drug-skin interactions^30^ may occur. Drug-CE interactions have effects on the rate and extent of release of drug from the solvent. CE-skin interactions either increase or decrease penetration of a drug across the skin.^31^ Drug-CE interaction may be explained by the solubility parameter. Higher drug-solvent interaction (or higher solubility of the drug in the solvent) will be evident if the solubility parameter difference between these two is low.^30^ However, if the drug molecule has a higher affinity for the CE it may remain preferentially in the CE and low permeation of the drug will be observed.^20^ Solvent-skin interaction will be discussed in section 3 of this article. Drug-skin interaction is mainly affected by the physicochemical properties (molecular weight, log P, melting point and solubility parameter) of the drug. Drug-solvent-skin interactions may be explained by the ‘push-pull’ effect (Figure 3).^32^

‘Push’ effects are of two types. If the solubility parameter difference between drug and CE is high, attraction of drug will be lower towards the CE and the drug will be easily escaped into the skin from the CE.^30,32^ Drugs having higher affinity for the CE, it will be held firmly by the CE and will not allow to penetrate through the SC.^20^Additionally, by increasing the thermodynamic activity, the drug will be pushed into the SC by the CE.^20,32,33^ The ‘pull’ effect explains that CEs change the SC by structural transformation and therefore, increase the solubility of the drug into the SC or drag the drug molecule while diffusing through the skin.^32,33^

### Modification of the SC to enhance drug penetration

There are physical and chemical methods to enhance the penetration of a drug in the skin. Physical enhancers involve iontophoresis, sonophoresis, phonophoresis, magnetophoresis, electroporation, thermophoresis, radiofrequency, needleless injection, microneedle etc. Both techniques involve alter the SC in such a way so that drug can penetrate the SC and reach the target site. Here, the effect of different classes of CE on skin will be discussed elaborately.

### Chemical enhancers (CEs)

‘CEs are pharmacologically inactive compounds which partition and diffuse into the skin and interact with SC components’. They are generally regarded as safe.^34^ CEs increase the permeation of drugs by interacting with the intracellular route, interacting in the intercellular route and by modifying the solubility or partition of the SC. In the intercellular route, the solute can interact with the polar head group,in the aqueous regions of intercellular bilayers and interacting in the lipid regions of intercellular bilayers. The skin penetration abilities of selected CEs are discussed below:

### Water

Water is the most common and safe penetration enhancer which is used for transporting both drug and cosmetic materials into the skin. Hydrating the skin or using moisturisers can be easiest way to deliver hydrophilic molecules effectively. The water content of the SC is usually 5 to 10%, which can be increased up to 50% under occlusive condition.^35^ In 1987, Barry reported that water molecule acts in both inter and intra-cellular pathways to enhance the permeation of both hydrophilic and lipophilic drugs.^36^ In case of intracellular region, in dry condition, the SC provides significant barrier to drug molecules because of the presence of several hydrogen bonding group. Since the SC becomes hydrated, the proteinaceous region takes up water. The arrangement of protein of that region becomes disordered and water starts competing for the hydrogen binding sites on the protein, and therefore, reduces the interaction between them. In this way, permeation of molecule through the intracellular pathway increases.^36,37^ Barry also stated that water molecule binds with the polar head group and forms a small hydration shell in the lipid bilayer region via hydrogen bonding. This leads to loosening of lipid packing and extending hydrophilic domain.^36^ However, later studies found that water does not cause a massive lipid disorder,^38^ it may cause slight disordering of a small population of the SC lipid.^39^ Water also found not to swell lipid bilayer but can be present in very small quantities in the polar head group of the lipid bilayer region.^18^ The excess amount of water the SC absorbs may be present in the corneocytes (intracellular region) or may be present as a separate phase in the intercellular region.^18,40^

### Alcohols

In short chain alcohols, ethanol is the most widely used and studied CE for skin drug delivery in topical and transdermal formulations. Ethanol is also used in such formulations to aid solubility of poorly water soluble drugs or as a cosolvent.^41^ At low concentration, ethanol displaces the bound water in the polar head group and disrupts the lipid-polar head/membrane interfacial region. This leads to increase in the interfacial area. At high concentration, ethanol extract lipid and proteins from the SC and thus forms pores in the SC0.^18,42^ Ethanol helps penetrating the drug by increasing the solubility in the formulation and by altering the solubility parameter of the SC towards the drug. The residence time of ethanol on skin is short due to its volatile nature.^43^ Therefore, higher thermodynamic activity of the drug dissolved in ethanol pushes the drug molecule into or across the SC. In addition, ethanol rapidly penetrates the SC by the mechanisms stated above, which also pulls the drug molecule along with it through the SC.^42^ Recently, Moghadam *et al.* reported no change in short and long lamellar spacing of the SC structure by ethanol. The authors suggested that the penetration enhancing property of ethanol might be due to the solvent’s ability to solubilise drug molecule into the SC.^44^

Fatty or long chain alcohol showed a parabolic relationship with the permeation of melatonin with the carbon chain length of saturated fatty alcohol. Melatonin permeation was found to increase up to chain length of 10 carbons (decanol) and then decreases. Decanol caused highest permeation enhancement for melatonin.^45^ Lipid extraction was the mechanism of enhancing the permeation of drug for D-hexanol and D-octanol. However, D-decanol was not found to disrupt the lipid content.^46^

### Amides

#### Azone

Azone (Laurocapram) is the first compound which was specially developed as a penetration enhancer.^35^ Azone contains a seven membered polar head group attached with its 12 carbon chain.^43^ It mainly reduces diffusional resistance of a drug into the SC.^43^ Because of this structure, it is inserted into the lipid bilayer region with the seven-membered polar group in the polar plane and the dodecyl chain in the lipid region. In this way, it disrupts the highly ordered lipid packing of the lipid bilayer.^18,35^ Azone has been found to increase permeation of hydrophilic, hydrophobic and some peptides. This CE is effective at low concentration (1 to 5%).^35^ Azone imparts its penetration enhancing property more efficiently in conjunction with propylene glycol (PG) rather than alone. Azone only modifies the intercellular region; however, PG acts in the intracellular pathway. Therefore, combination of these two CEs efficiently delivered a number of drug molecule.^36^ Although Azone has been investigated as a CE for over 25 years,^35^ still it is not used in any commercial formulation.^43^

### Esters

#### Alkyl and benzoic acid esters

Ethyl acetate has been found to increase permeation of levonorgestrel. However, its exact mechanism of action is not confirmed yet.^43^ Another ester compound, octyl salicylate (OS) is used as a sunscreen at a concentration up to 5%. OSAL is also found to enhance the penetration of fentanyl and testosterone.^43,47,48^ Being a lipophilic solvent, OS previously found to alter the highly ordered lipid bilayer region of the SC converting the gel phase of the lipid lamellae to a liquid phase. However, recent studies did not reveal any lipid distortion result in the SC.^49,50^ It has been postulated that OS remains in the lipid gel phase as a ‘pool’ rather than interacting with the lipids and therefore enhance the diffusivity of the compound.^43^

#### Fatty acid esters

This group includes isopropyl myristate (IPM), propylene glycol monocaprylate (PGMC), Propyleneglycolmonolaurate (PGML).

#### IPM 

IPM is the most widely investigated fatty acid ester. IPM found to impart fluidisation and hampers the order of lipid lamellae. However, later it was reported that IPM inserted into lipophilic region anchoring its isopropyl group in the polar region and hence interact with the lipid region.^51^ In a recent study it has been reported that because of its branched structure and highly mobile terminal isopropyl group, IPM did not mix with other SC lipid. This is why IPM perturbed and disordered the assembly of lipid lamellae.^52^ IPM was also found to cause phase segregation and lipid extraction from the SC.^52^ In a very recent study, neat IPM was found to be present in higher quantities in the skin and therefore, aided higher retention of anthramycin in the skin rather than permeation.^53^

PGMC is the fatty acid ester which showed enhanced drug permeation alone^54-57^sometimes and mostly in combination with diethyleneglycolmonoethyl ether (Transcutol®, TC).^58,59^ However, it was found to deposit inside the SC, retaining higher quantities of drug.^60^ Recently, Haque *et al.* sowed that similar to IPM, PGMC retains in the skin in higher quantities which also helps to retain higher quantities of drug dissolve in it.^53^ Takahashi *et al.* found that PGMC reduces the resistance to drug diffusion across the skin by interfering the SC lipid packing. Whereas, no extraction of lipid was evident.^60^ Moghimipur *et al.* found contradicting results on mechanism of action of PGMC. Fourier transform infrared spectroscopy (FTIR) studies showed SC lipid extraction or fluidisation by PGMC. On the other hand, Differential Scanning Calorimetry (DSC) indicated bilayer cohesion by PGMC. Because of the opposite effects, authors concluded that penetration enhancement effect of PGMC was low.^55^ Moghimipur *et al.* also reported that PGMC interacted mostly with the SC keratins and modifies the skin lipid.^55^

#### PGML

PGML is recently used to delivery drug percutaneously in transdermal formulation. Like PGMC, PGML found to increase drug penetration.^59,61^ Haque *et al*. showed that when anthramycin was applied on human skin in pure PGML, retention of small amount of PGML in the skin enhanced the drug permeation significantly.^53^ However, much enhanced permeation was observed along with hydrophilic enhancers, such as propylene glycol (PG) and TC.^62-65^ Parisi *et al.* showed that combining PGML with PG in 50:50 ratio increased the skin retention of hexamidinediisethionate in significant quantities.^66^ The mechanism of action of the PGML is not clearly stated in the literature. However, it may work in the similar way as PGMC.

### Ether alcohol

#### Transcutol® (TC)

TC is a hydrophilic CE with the similar solubility parameter as the skin [10.62 (cal/cm^3^)^1/2^].^67^ TC is used as a penetration enhancer in both topical and transdermal formulations. TC has been found to increase the flux and retention of drugs.^33,68-71^ Recently, Haque *et al.* showed that TC as a solvent penetrated and retained in the human skin in highest quantities compared with other selected hydrophilic solvents. Therefore, a moderate amount of drug was permeated and accumulated in the skin with the help of TC. TC clearly showed ‘pull’ effect aiding higher absorption of drug molecule.^53^ The main mechanism of this solvent to enhance permeation is to increase the partition parameter of the drug into the skin. This may be because of the close solubility parameter of TC with skin. TC has been reported to present inside the SC as intraceutaneous depot. TC being a hydrophilic molecule, is inserted into the aqueous region between the polar head group and induce swelling of the bilayer region without altering the bilayer structure. Therefore, the swollen lipids hold the drugs soluble in the SC. In this way, TC aids to accumulate drugs in the SC (Pull effect).^68,72,73^ Due to its hydrophilicity and hydrophobicity, it was suggested to interact with the intracellular lipids of the other layers of epidermis and dermis. The barrier function of the SC was not altered by TC.^72^ However, recently Moghadam *et al.* suggested that slight disorder in the lamellar structure of the SC caused by TC, which leads to membrane fluidity.^44^ Caon *et al*. showed that skin permeation of ioniaside in TC was reduced but skin retention was increased and the statement goes well with the ‘ranscutaneous depot’ theory. The authors also conducted DSC and FTR experiments with rat skin after the permeation study. DSC analysis showed that skin lipid disruption was not caused by TC but slight membrane fluidisation. FTR analysis also confirmed that TC increased the order of both lipid and protein domains of the skin.^74^

### Fatty acids

#### Oleic acid (OA)

OA is an unsaturated lipophilic C fatty acid that is commonly associated with enhanced penetration of polar to fairly polar molecules.^75^ OA has been used in both topical and transdermal formulations due to its desirable properties. OA causes temporary and reversible disruption of the SC lipids, increasing fluidisation and diffusivity of the skin. This theory was reinforced in a study using spectrometric and calorimetric measurements which showed that OA increased lipid fluidity and permeant flux in porcine skin.^76^ Due to the bent structure of OA, it disrupts the ordered orientation of lipid region and increase the fluidity. The mechanism of action of OA is similar to Azone. However, OA did not found to disturb the structure very drastically.^36^ More specifically, the kinked nature of OA (bent *cis* configuration) has mainly been associated with the separation of SC lipid regions, which reduces barrier function of the SC. However, despite the advantages of OA, dermal side effects of unsaturated fatty acids have been reported.^77^ These can be overcome by modification of the carboxylic terminal, which reduces the acidic nature of the fatty acid allowing safe use.^78^

The existence of OA as a separate phase within SC lipids was revealed in a study conducted on porcine SC using Fourier Transform Infrared spectroscopy (FT-IR). Results showed that OA interacted with the SC lipids by reducing the lipid transition temperature (T_m_), and by increasing the “conformational freedom of lipid alkyl chains” higher than their T_m_. OA may cause some sort of permeable defect within the SC that enhances diffusion of permeants. This leads to increased permeability^75^ and diffusion coefficient in Fick’s law. More recently, a study was conducted using urea, caffeine and diclofenac sodium, in the presence of OA as a CE. In that study OA showed a significant effect on the SC, especially in the model membrane with the higher ratio of phytosphingosine-based ceramide.^79^ Recently, Atef *et al.* showed a time dependent enhancement of OA permeation in rat skin in terms of spectral change in Raman Spectroscopy.^80^

### Glycols

#### Propylene glycols (PG)

PG has been used as a cosolvent in topical and transdermal products since long. It is a well-established topical CE. It acts as a CE not only by on its own but also in combination with a number of other CEs. PG mainly increases drug permeation by improving the partition properties of drugs in to the SC. It solvates the α-keratin and therefore reduce drug-tissue binding.^36^ Bouwstra *et al.* conducted Small-angle X-ray scattering (SAXS) and differential thermal analysis (DTA) of the SC after pre-treating with PG. DTA results showed that PG interacted with the SC lipid. On the other hand, SAXS showed that SC lipids were unaffected by PG. Due to these two contradicting findings, Bouwstra *et al.* concluded that PG being a hydrophilic molecule, incorporated into the polar head group of the lipid bilayer. Therefore, it increased mean interfacial area per lipid molecule. PG also induced lateral swelling (side by side). In order to compensate the lateral swelling (to maintain the density of the alkyl chain region), the chain length of the lipids were decreased. Therefore, no change in the SAXS was observed after pre-treatment with PG.^81^ Recently, Moghadam *et al.* and Furuishi *et al.* conducted several experiments on PG and found no significant skin lipid alteration by PG.^44,82^ Therefore, Moghadam *et al.* suggested improvement of skin partitioning is the main mechanism of PG to enhance permeation.^44^ In addition, Mohammed *et al.* showed that PG increased transepidermal water loss (TWEL) and KLK 7 protease activity in the skin, therefore reduce the barrier property of the SC.^83^ PG was shown to be less penetrating molecule to the human skin compared with TC. In case of hydrophilic molecule, it imparts its skin penetration effects by both ‘push’ and ‘pull’ effects.^53^ Atef *et al*. showed that in Raman Spectrum, after applying PG on rat skin, the intensity of PG peak (at 840 cm-1) decreased with time in comparison with the skin peaks. The authors concluded that the decrease in PG peak intensity is the indication of increased diffusion of PG in the skin.^80^

### Pyrrolidones

#### N-methyl-2-pyrrolidone (NMP) and 2-pyrrolidone (2P)

NMP and 2P are the pyrrolidones which have been investigated mostly for years.^42^ Both of the CEs are dissolved in water in all proportions. The CEs found to enhance the permeation of both hydrophilic and lipophilic compounds.^42^ DSC studies conducted by Barry showed that these two CEs partition in the corneocyte region (intracellular region) at low concentration and affect the intercellular region at high concentration. These molecules produces a solvation shell around the polar head group, loosens the tight packing of the lipid bilayer and induce lipid fluidity.^36^ However, Trommer *et al.* suggested that comparatively hydrophilic pyrrolidones work by acting on the polar region and hydrophobic pyrrolidones (such as NMP) work on the lipophilic region.^35^ However NMP was found to cause erythema, swelling, skin irritation, thickness of skin etc.^84^ In addition, the clinical use of these molecules was restricted due to its skin cytotoxic properties.^43^

### Sulphoxides

#### Dimethyl sulphoxides (DMSO)

DMSO is an aprotic solvent. Because of its special structure, it has broad solvent properties. Like pyrrolidones, DMSO interacts with keratin when applied in low concentration (20%). Due to its small molecular size, the compound can easily penetrate the region of protein subunit. DMSO then displaces the protein-water and hampers the native configuration of the protein (by interfering with hydrogen bonding and hydrophobic interactions). Therefore, drug/compounds get sufficient loose or flexible areas to penetrate through the SC. However, skin’s impermeable characteristics restores immediately after removing DMSO as the solvent passes out the skin very quickly and gradual removal of protein-DMSO by competitive bonding with cellular water.^36^ In the intercellular region, at higher concentration (above 60%), DMSO produces a large solvation shell around the lipid polar head group by displacing water from the polar group. Hence, it loosens the lipid packing more severely compared with water. This leads to increase the aqueous region in the intercellular pathway and helps to promote the permeation of hydrophilic compounds.^36^ In a recent study, DMSO was found to change the highly ordered gel phase of Ceremide 2 into loosely packed liquid crystalline phase at greater than 0.4 mole fraction concentration. It has been also shown that DMSO replaced the water from the interface at higher concentration. It also induced lateral swelling by increasing area per lipid in the lipid bilayer region.^85^

### Surfactants

Topical and transdermal formulation contains surfactants in order to increase the solubility of a compound in the formulation. Sodium lauryl or dodecyl sulphate (SLS) (anionic surfactant). Anionic surfactants, such as SLS acts on the skin by affecting both intra- and intercellular pathways.^36^ For this reason, irritation and skin damage are very common with this type of surfactant.^35,43^ SLS swells the SC and the swollen keratin can absorb more water and help to penetrate drug molecule. Additionally, SLS also unfolds and extends the alpha keratin and opens up the polar pathway for permeating the drug molecule.^36^ A recent study revealed that SLS interacts with lipids and is incorporated there to create a lamellar structure.^44^

The cationic surfactants include amines, alkylimidazolines, alkoxylated amines, and quaternary ammonium compounds (or Quats). Cationic surfactants disorders SC lipid organisation more drastically than anionic and non-ionic surfactant. It mainly affects the lateral packing of the SC lipid.^44^ Therefore, molecules of this group are more effective as chemical enhancers than anionic and non-ionic ones.^35^ However, as this group cause skin irritation, further use in dermal formulation is not encouraged.^43^

The primary non-ionic surfactants used for cosmetics include alcohols (cetyl or stearyl alcohol), alkanolamides, esters, and amine oxides. Non-ionic surfactants disordered the SC less radically compared with ionic surfactants.^44^ This is why, these surfactants are less irritating to the skin and comparatively less effective as penetration enhancer than the other types and therefore, regarded as safe.^42^

### Terpenes

Terpenes are found in essential oils.^42^ These are lipophilic compounds which mainly act on the lipid pathway of the SC. Since it produces less skin irritation, it is regarded as safe.^35^ Smaller terpenes are found to enhance drug permeation more effectively than larger terpenes. Furthermore, polar terpenes (menthol, 1,8-cineole, etc.) improve the penetration of hydrophilic and non-polar terpenes (D-limolene) improve the penetration of hydrophobic molecules in the SC. D-limolene, 1,8 cineole hampers the arrangement of the SC lipid. On the other hand, nerolidol, a sesquiterpene strengthens the lipid bilayer may be by incorporating into the bilayer.^42^ However, Moghadam *et al.* investigated different terpenes and terpenoids and found that nerol disrupted the lipid lamellae more drastically than other types of molecules. The hydroxyl group and the alkene of the molecule may donate hydrogen bond and disrupt the interaction between the existing hydrogen bond between the ceramide groups of the lipid bilayer.

### Phospholipids

The use of phospholipids as penetrations enhancer has been widely studied as a vesicle or liposomes. However, there are fewer studies of this molecule as non-vesicular chemical enhancers. Because of its lipophilic characteristics, it occludes the SC and increase the hydration of the skin. Therefore, penetration of a drug molecule is enhanced. As a vesicle it is incorporated or fused into the SC lipid and liberates the molecule in the solvent in which it was poorly soluble. Thus thermodynamic activity as well as drug permeation were increased.^42^

### Cyclodextrines

Cyclodextrins are not typical enhancers as they cannot penetrate the SC. They form inclusion complexes of hydrophobic drugs and improve their aqueous solubility. However, several studies showed penetration enhancement of certain drugs when cyclodextrin was used in combination with lipophilic enhancers (fatty acids, azone etc.).^35^

## Discussion

The various sites of action of different types of CE are as follows:

The intracellular route is the polar route where keratin fibrils are present. Aprotic solvents [for example, dimethyl sulfoxide (DMSO)], pyrrolidone, surfactants interact with the keratin and may disrupt the ordered arrangement in corneocytes. Openings may be formed because of the extensive interactions. Therefore, higher permeability coefficients and fluxes can be observed.^24^

In the intercellular region, CEs may act in three ways. By interaction with the polar head groups, they modify the hydrogen-bonding and ionic forces. As a result, the packing order of the polar head group in the aqueous region may be distressed. This disturbance fluidises the lipid region which also allows polar CEs to diffuse into the aqueous region and increase the volume of water between the lipid layers.^34^

Some CEs act directly in the aqueous region present between the polar head groups. CEs or solvents [for example, propylene glycol (PG), DMSO, Transcutol® (TC), ethanol (EtOH), pyrrolidones] increase the solubilising property (or change the solubility parameter) of this region so that drug can partition readily into the SC. CEs also act within the alkyl chain (lipid region) of the lipid bilayer by disturbing the lipid packing and enhancing the fluidity of the lipid chains. There are some CEs (DMSO, alcohols, etc.) which may also cause lipid extraction.^24,34^Table 1 summarises the site of action of various types of CE in skin.

**Table 1:** A summary of reported mechanism of action of commonly used topical and transdermal CEs

**Site of action**	**CE**
Interact with corneocytes or keratin	Water^36,37^, PGMC^55^, NMP, 2P^36^, DMSO^36^, SLS^36^
Near polar head groups of the lipid bilayer region	Water^18,36^, ethanol (at low concentration), NMP, 2P^36^, DMSO^36,85^
Extraction of intercellular lipids	Ethanol (high concentration)^18,42^, D-hexanol and D-octanol^46^, IPM^52^
Disruption of highly ordered lipid packing of SC bilayer region	Azone^18,35^, OS^49,50^, IPM^52^, PGMC^60^, OA^77^, DMSO^85^, Nerol^44^
Present as a ‘pool’ in the lipid region of the bilayer structure of the SC	OS^43^ Phospholipids^42^
Phase separation	IPM^52^
Creates a lamellar structure after incorporating into the lipid phase	SLS^44^
Slight disordering in the lamellar arrangement of the SC	TC^44^
Increase the solubility or partitioning parameter of the drug into the SC	Ethanol^44^, TC, PG^36,44^

## Conclusion

Several approaches have been taken to enhance penetration of drug molecules across the SC. However, chemical enhancers have been found to be the most efficient and simplest ones. In addition, the chemical enhancers improve the permeation of the molecules in the skin in a most cheap and effective way. Except for some established CEs, the in depth mechanism of action of most of the CEs are poorly understood until now. In this article, we have summarized the studies conducted till now on different CEs. CEs induce structural transformations and enhance drug permeation across the SC by interacting with the major permeation pathway (intercellular region). Some CEs or combination of CEs show dual action, that is, by altering the partition parameter of the skin and by interacting with either the intra-or intercellular regions. ‘Pull- Effect’ is the result of penetration enhancing properties of either a single of combined CE(s). The mechanism of action of some CEs in molecular levels has been included in this article as well. Though, in case of some CEs contradicting mechanism of actions were suggested, the information would still be a basis to do further studies to confirm the exact mechanism of action. Still we need further studies to have a concrete understanding on the CEs. However, the summarized information on CEs would be useful for the formulation scientists to develop simple topical and transdermal formulations with improved permeation or penetration of a compound.

## Ethical Issues

Not applicable

## References

[1] Haque T, Crowther JM, Lane ME, Moore DJ (2016). Chemical ultraviolet absorbers topically applied in a skin barrier mimetic formulation remain in the outer stratum corneum of porcine skin. Int J Pharm.

[2] D’Arcy Y. Targeted topical analgesics for acute pain. PainMedicine News; 2015 [cited 2016 25 November]; Available from: http://www.painmedicinenews.com/Review-Articles/Article/12-14/Targeted-Topical-Analgesics-For-Acute-Pain/28992/ses=ogst.

[3] Güngör S, Erdal MS, Aksu B (2013). New formulation strategies in topical antifungal therapy. J Cosmet Dermatol Sci Appl.

[4] McGrath JA, Eady RAJ, Pope FM. Anatomy and organization of human skin. In: Burns T, Breathnach S, Cox N, Griffiths C, editors. Rook's textbook of dermatology. 7th ed. Blackwell Publishing, Inc.; 2008. p. 45-128.

[5] Katz M, Poulsen BJ. Absorption of drugs through the skin. In: Brodie BB, Gillete J, editors. Handbook of experimental pharmacology. Berlin, Heidelberg, New York: Springer- Verlag; 1971. p. 103-74.

[6] Wood EJ, Bladon PT (1985). The human skin.

[7] Benson HAE. Skin structure, function, and permeation. In: Benson HAE, Watkinson AC, editors. Topical and transdermal drug delvery: Principles and practice. New Jersey: Jhon Wiley & Sons, Inc.; 2012. p. 3-22.

[8] Christophers E (1971). Cellular architecture of the stratum corneum. J Invest Dermatol.

[9] Elias PM, Cooper ER, Korc A, Brown BE (1981). Percutaneous transport in relation to stratum corneum structure and lipid composition. J Invest Dermatol.

[10] Michaels AS, Chandrasekaran SK, Shaw JE (1975). Drug permeation through human skin: Theory and in vitro experimental measurement. Aiche J.

[11] Elias PM (1988). Structure and function of the stratum corneum permeability barrier. Drug Dev Res.

[12] Wertz PW, Downing DT. Stratum corneum: Biological and biochemical considerations. In: Hadgraft J, Guy RH, editors. Transdermal drug delivery: Developmental issue and research initiatives. New York: Marcel Dekker, Inc.; 1989. p. 1-22.

[13] Bouwstra J, Pilgram G, Gooris G, Koerten H, Ponec M (2001). New aspects of the skin barrier organization. Skin Pharmacol Appl Skin Physiol.

[14] Pilgram GSK, Pelt AME-v, Bouwstra JA, Koerten HK (1999). Electron diffraction provides new information on human stratum corneum lipid organization studied in relation to depth and temperature. J Invest Dermatol.

[15] Golden GM, Guzek DB, Kennedy AE, McKie JE, Potts RO (1987). Stratum corneum lipid phase transitions and water barrier properties. Biochem.

[16] Blank IH (1952). Factors which influence the water content of the stratum corneum. J Invest Dermatol.

[17] Swartzendruber DC, Wertz PW, Kitko DJ, Madison KC, Downing DT (1989). Molecular models of the intercellular lipid lamellae in mammalian stratum corneum. J Invest Dermatol.

[18] Suhonen TM, Bouwstra JA, Urtti A (1999). Chemical enhancement of percutaneous absorption in relation to stratum corneum structural alterations. J Control Release.

[19] Potts RO, Francoeur ML (1991). The influence of stratum corneum morphology on water permeability. J Invest Dermatol.

[20] Higuchi T (1960). Physical chemical analysis of percutaneous absorption process from creams and ointments. J Soc Cosmet Chem.

[21] Scheuplein RJ, Blank IH (1971). Permeability of the skin. Physiol Rev.

[22] Morrow DIJ, McCarron PA, Woolfson AD, Donnelly RF (2007). Innovative strategies for enhancing topical and transdermal drug delivery. Open Drug Deliver J.

[23] Scheuplein HJ, Blank IH, Brauner GJ, MacFarlane DJ (1969). Percutaneous absorption of steroids. J Invest Dermatol.

[24] Benson HAE (2005). Transdermal drug delivery: Penetration enhancement techniques. Curr Drug Deliv.

[25] Hueber F, Wepierre J, Schaefer H (1992). Role of transepidermal and transfollicular routes in percutaneous absorption of hydrocortisone and testosterone: In vivo study in the hairless rat. Skin Pharmacol.

[26] Albery WJ, Hadgraft J (1979). Percutaneous absorption: In vivo experiments. J Pharm Pharmacol.

[27] Potts RO, Guy RH (1992). Predicting skin permeability. Pharm Res.

[28] Nemanic MK, Elias PM (1980). In situ precipitation: A novel cytochemical technique for visualization of permeability pathways in mammalian stratum corneum. J Histochem Cytoche.

[29] Boddé HE, van den Brink I, Koerten HK, de Haan FHN (1991). Visualization of in vitro percutaneous penetration of mercuric chloride; transport through intercellular space versus cellular uptake through desmosomes. J Control Release.

[30] Roberts MS, Cross SE, Pellett MA. Skin transport. In: Walters KA, editor. Dermatological and transdermal formulation. New York, Basel: Marcel Dekker, Inc.; 2002. p. 89-183.

[31] Benson HA, Sarveiya V, Risk S, Roberts MS (2005). Influence of anatomical site and topical formulation on skin penetration of sunscreens. Ther Clin Risk Manag.

[32] Kadir R, Stempler D, Liron Z, Cohen S (1987). Delivery of theophylline into excised human skin from alkanoic acid solutions: A “push-pull” mechanism. J Pharm Sci.

[33] Mura P, Faucci MT, Bramanti G, Corti P (2000). Evaluation of transcutol as a clonazepam transdermal permeation enhancer from hydrophilic gel formulations. Eur J Pharm Sci.

[34] Lane ME, Santos P, Watkinson AC, Hadgraft J. Passive skin permeation enhancement. In: Benson HAE, Watkinson AC, editors. Topical and transdermal drug delivery principle and practice. New Jersey: Wiley-Blackwell; 2012. p. 24-42.

[35] Trommer H, Neube RHH (2006). Overcoming the stratum corneum: The modulation of skin penetration. Skin Pharmacol Physiol.

[36] Barry BW (1987). Mode of action of penetration enhancers in human skin. J Control Release.

[37] Gwak HS, Oh IS, Chun IK (2004). Transdermal delivery of ondansetron hydrochloride: Effects of vehicles and penetration enhancers. Drug Dev Ind Pharm.

[38] Mak VW, Potts R, Guy R (1991). Does hydration affect intercellular lipid organization in the stratum corneum?. Pharm Res.

[39] Gay CL, Guy RH, Golden GM, Mak VH, Francoeur ML (1994). Characterization of low-temperature (i.E., <65 degrees c) lipid transitions in human stratum corneum. J Invest Dermatol.

[40] Van Hal DA, Jeremiasse E, Junginger HE, Spies F, Bouwstra JA (1996). Structure of fully hydrated human stratum corneum: A freeze-fracture electron microscopy study. J Invest Dermatol.

[41] Trommer H, Neubert RH (2006). Overcoming the stratum corneum: The modulation of skin penetration. A review. Skin Pharmacol Physiol.

[42] Williams AC, Barry BW (2004). Penetration enhancers. Adv Drug Deliv Rev.

[43] Lane ME (2013). Skin penetration enhancers. Int J Pharm.

[44] Moghadam SH, Saliaj E, Wettig SD, Dong C, Ivanova MV, Huzil JT (2013). Effect of chemical permeation enhancers on stratum corneum barrier lipid organizational structure and interferon alpha permeability. Mol Pharm.

[45] Andega S, Kanikkannan N, Singh M (2001). Comparison of the effect of fatty alcohols on the permeation of melatonin between porcine and human skin. J Control Release.

[46] Dias M, Naik A, Guy RH, Hadgraft J, Lane ME (2008). In vivo infrared spectroscopy studies of alkanol effects on human skin. Eur J Pharm Biopharm.

[47] Santos P, Watkinson AC, Hadgraft J, Lane ME (2011). Formulation issues associated with transdermal fentanyl delivery. Int J Pharm.

[48] Santos P, Watkinson AC, Hadgraft J, Lane ME (2012). Influence of penetration enhancer on drug permeation from volatile formulations. Int J Pharm.

[49] Casal HL, Mantsch HH (1984). Polymorphic phase behaviour of phospholipid membranes studied by infrared spectroscopy. Biochim Biophys Acta.

[50] El Maghraby GM, Campbell M, Finnin BC (2005). Mechanisms of action of novel skin penetration enhancers: Phospholipid versus skin lipid liposomes. Int J Pharm.

[51] Brinkmann I, Muller-Goymann CC (2005). An attempt to clarify the influence of glycerol, propylene glycol, isopropyl myristate and a combination of propylene glycol and isopropyl myristate on human stratum corneum. Pharmazie.

[52] Engelbrecht TN, Deme B, Dobner B, Neubert RH (2012). Study of the influence of the penetration enhancer isopropyl myristate on the nanostructure of stratum corneum lipid model membranes using neutron diffraction and deuterium labelling. Skin Pharmacol Physiol.

[53] Haque T, Rahman KM, Thurston DE, Hadgraft J, Lane ME (2017). Topical delivery of anthramycin i. Influence of neat solvents. Eur J Pharm Sci.

[54] Takahashi K, Komai M, Kinoshita N, Nakamura E, Hou XL, Takatani-Nakase T (2011). Application of hydrotropy to transdermal formulations: Hydrotropic solubilization of polyol fatty acid monoesters in water and enhancement effect on skin permeation of 5-fu. J Pharm Pharmacol.

[55] Moghimipour E, Salimi A, Zadeh BSM (2013). Effect of the various solvents on the in vitro permeability of vitamin b12 through excised rat skin. Trop J Pharm Res.

[56] Gwak HS, Kim SU, Chun IK (2002). Effect of vehicles and enhancers on the in vitro permeation of melatonin through hairless mouse skin. Arch Pharm Res.

[57] Lee J, Chun I (2012). Effects of various vehicles and fatty acids on the skin permeation of lornoxicam. J Pharm Invest.

[58] Gwak HS, Chun IK (2002). Effect of vehicles and penetration enhancers on the in vitro percutaneous absorption of tenoxicam through hairless mouse skin. Int J Pharm.

[59] Cho YA, Gwak HS (2004). Transdermal delivery of ketorolac tromethamine: Effects of vehicles and penetration enhancers. Drug Dev Ind Pharm.

[60] Takahashi K, Sakano H, Yoshida M, Numata N, Mizuno N (2001). Characterization of the influence of polyol fatty acid esters on the permeation of diclofenac through rat skin. J Control Release.

[61] Lee KE, Choi YJ, Oh BR, Chun IK, Gwak HS (2013). Formulation and in vitro/in vivo evaluation of levodopa transdermal delivery systems. Int J Pharm.

[62] Kim KH, Gwak HS (2011). Effects of vehicles on the percutaneous absorption of donepezil hydrochloride across the excised hairless mouse skin. Drug Dev Ind Pharm.

[63] Jung SY, Kang EY, Choi YJ, Chun IK, Lee BK, Gwak HS (2009). Formulation and evaluation of ubidecarenone transdermal delivery system. Drug Dev Ind Pharm.

[64] Choi JS, Cho YA, Chun IK, Jung SY, Gwak HS (2007). Formulation and evaluation of ketorolac transdermal systems. Drug Deliv.

[65] Chiang C-M, Cleary GW, inventors; Cygnus Therapeutic Systems, assignee. Skin permeation enhancer compositions, and methods and transdermal systems associated herewith patent 5,053,227. 1991.

[66] Parisi N, Paz-Alvarez M, Matts PJ, Lever R, Hadgraft J, Lane ME (2016). Topical delivery of hexamidine. Int J Pharm.

[67] Liron Z, Cohen S (1984). Percutaneous absorption of alkanoic acids ii: Application of regular solution theory. J Pharm Sci.

[68] Chadha G, Sathigari S, Parsons DL, Jayachandra Babu R (2011). In vitro percutaneous absorption of genistein from topical gels through human skin. Drug Dev Ind Pharm.

[69] Puglia C, Bonina F, Trapani G, Franco M, Ricci M (2001). Evaluation of in vitro percutaneous absorption of lorazepam and clonazepam from hydro-alcoholic gel formulations. Int J Pharm.

[70] Prasanthi D, Lakshmi PK (2012). Effect of chemical enhancers in transdermal permeation of alfuzosin hydrochloride. ISRN Pharm.

[71] Shah PP, Desai PR, Patlolla R, Klevans L, Singh M (2014). Effect of combination of hydrophilic and lipophilic permeation enhancers on the skin permeation of kahalalide f. J Pharm Pharmacol.

[72] Panchagnula R, Ritschel WA (1991). Development and evaluation of an intracutaneous depot formulation of corticosteroids using transcutol as a cosolvent: In-vitro, ex-vivo and in-vivo rat studies. J Pharm Pharmacol.

[73] Ritschel WA, Panchagnula R, Stemmer K, Ashraf M (1991). Development of an intracutaneous depot for drugs. Binding, drug accumulation and retention studies, and mechanism of depot. Skin Pharmacol.

[74] Caon T, Campos CE, Simoes CM, Silva MA (2015). Novel perspectives in the tuberculosis treatment: Administration of isoniazid through the skin. Int J Pharm.

[75] Ongpipattanakul B, Burnette RR, Potts RO, Francoeur ML (1991). Evidence that oleic acid exists in a separate phase within stratum corneum lipids. Pharm Res.

[76] Golden GM, McKie JE, Potts RO (1987). Role of stratum corneum lipid fluidity in transdermal drug flux. J Pharm Sci.

[77] Sintov A, Ze'evi A, Uzan R, Nyska A (1999). Influence of pharmaceutical gel vehicles containing oleic acid/sodium oleate combinations on hairless mouse skin, a histological evaluation. Eur J Pharm Biopharm.

[78] Ben-Shabat S, Baruch N, Sintov AC (2007). Conjugates of unsaturated fatty acids with propylene glycol as potentially less-irritant skin penetration enhancers. Drug Dev Ind Pharm.

[79] Ochalek M, Podhaisky H, Ruettinger HH, Neubert RH, Wohlrab J (2012). Sc lipid model membranes designed for studying impact of ceramide species on drug diffusion and permeation, part iii: Influence of penetration enhancer on diffusion and permeation of model drugs. Int J Pharm.

[80] Atef E, Altuwaijri N (2018). Using raman spectroscopy in studying the effect of propylene glycol, oleic acid, and their combination on the rat skin. AAPS PharmSciTech.

[81] Bouwstra JA, de Vries MA, Gooris GS, Bras W, Brussee J, Ponec M (1991). Thermodynamic and structural aspects of the skin barrier. J Control Release.

[82] Furuishi T, Kato Y, Fukami T, Suzuki T, Endo T, Nagase H (2013). Effect of terpenes on the skin permeation of lomerizine dihydrochloride. J Pharm Pharm Sci.

[83] Mohammed D, Hirata K, Hadgraft J, Lane ME (2014). Influence of skin penetration enhancers on skin barrier function and skin protease activity. Eur J Pharm Sci.

[84] Åkesson B (2001). Concise international chemical assessment document 35: N-methyl-2-pyrrolidone.

[85] Notman R, den Otter WK, Noro MG, Briels WJ, Anwar J (2007). The permeability enhancing mechanism of dmso in ceramide bilayers simulated by molecular dynamics. Biophys J.

